# Spatial Distribution of Pyrethroid Resistance and kdr Mutations in *Aedes aegypti* from La Guajira, Colombia

**DOI:** 10.3390/insects14010031

**Published:** 2022-12-29

**Authors:** Ronald Maestre-Serrano, Zulibeth Flórez-Rivadeneira, Juan M. Castro-Camacho, Eva Soto-Arenilla, Doris Gómez-Camargo, Paula Pareja-Loaiza, Gustavo Ponce-Garcia, Alan E. Juache-Villagrana, Adriana E. Flores

**Affiliations:** 1 Facultad de Ciencias de la Salud, Universidad Libre Seccional Barranquilla, Km 7 Antigua Via Puerto Colombia, Barranquilla 080001, Colombia; 2 Secretaria de Salud Departamental, Gobernacion de La Guajira, Calle 12 # 8-19, Riohacha 440001, Colombia; 3 Facultad de Medicina—Sede Zaragocilla, Universidad de Cartagena, Calle 30 N° 48-152, Cartagena de Indias 130001, Colombia; 4 Facultad de Ciencias de la Salud, Universidad Simon Bolivar, Carrera 59 No. 59-92, Barranquilla 080002, Colombia; 5 Facultad de Ciencias Biologicas, Universidad Autonoma de Nuevo Leon, Av. Universidad s/n Cd. Universitaria, San Nicolas de los Garza, NL 66455, Mexico

**Keywords:** *Aedes aegypti*, pyrethroids, kdr mutations

## Abstract

**Simple Summary:**

Current strategies to suppress arbovirus outbreaks include insecticide use against larvae and adult mosquitoes. The control of *Aedes aegypti* by insecticides is challenging due to a rapid increase in resistance. In Colombia, pyrethroids have been one of the most widely used insecticides to control adult forms of *Ae. aegypti* because of its low impact on the environment, low toxicity to mammals, and greater effectiveness. We detected the frequency and intensity of resistance to permethrin, deltamethrin, lambda-cyhalothrin, and associated kdr mutations in *Ae. aegypti* from La Guajira, Colombia. Modeling the spatial distribution of pyrethroid resistance and the mechanisms (e.g., kdr mutations) provoking it could enhance current national and departmental vector control programs by classifying areas according to the insecticide resistance status of *Ae. aegypti* populations and orient strategies such as rotations in endangered areas. Thus, benefits such as susceptibility recovery might be obtained.

**Abstract:**

Dengue, chikungunya, and Zika are of great concern to the public health of Colombia. One of the main control strategies for these diseases is the application of insecticides directed at the *Aedes aegypti* vector. However, insecticide resistance has been increasingly recorded in the country, making control measures difficult. Here, we evaluated the resistance profiles for pyrethroids in populations of *Ae. aegypti* from La Guajira, Colombia. The frequency (diagnostic dose, DD) and intensity (2×, 5×, and 10× DD) of resistance to permethrin, deltamethrin, and lambda-cyhalothrin were determined in 15 populations of *Ae. aegypti* from La Guajira, Colombia, using the bottle bioassay. The kdr mutations V1016I, F1534C, and V410L, were identified, and their allele and genotype frequencies were calculated. Finally, the mortality values for the analyzed pyrethroids were interpolated following the IDW method for predicting pyrethroid resistance. The populations of *Ae. aegypti* showed a high frequency of resistance to permethrin with a low to moderate intensity, which was associated with the triple-resistant haplotype LL410/II1016/CC1534. They remain susceptible to deltamethrin and, in some populations, expressed the risk of developing resistance to lambda-cyhalothrin.

## 1. Introduction

*Aedes aegypti* (Diptera: Culicidae) is a species of interest to public health as it is a competent vector of arboviral diseases such as dengue, Zika, yellow fever, and chikungunya, causing morbidity and mortality impacts worldwide [1]. In the Americas between the year 2020 and the epidemiological week 22 of 2022, 4,131,433 cases of arbovirosis were recorded, of which 3,883,586 corresponded to dengue cases, 221,735 cases of chikungunya and 26,112 cases of Zika [2,3,4,5]. Colombia is one of the countries that contributes the most to the incidence of these arboviral diseases in the Americas; between 1995 and 2019, 1,371,722 cases of dengue were recorded in the country, of which 252,623 cases (18.4%) were in the Caribbean region [6,7,8,9,10], with 4910 cases (1.9%) being reported in the department of La Guajira, which in the last decade has shown an upward trend in the incidence of this disease [11].

On the other hand, the first case of chikungunya reported in Colombia was identified in week 37 of 2014 [12]. From then until 2019, 489,528 cases of this disease have been reported in the country, with 119,104 cases (24.3%) occurring in the Caribbean region and 11,364 (9.5%) in La Guajira. The first outbreak of Zika in the country was reported in October 2015. From then until the epidemiological close of 2019, 109,999 cases were recorded in the country, of which 19,662 cases (17.8%) corresponded to the Caribbean region and 706 (3.5%) of these were for the department of La Guajira [13].

In the absence of a vaccine available for these arboviruses, control measures have been aimed at *Ae. aegypti*, such as physical control to eliminate domestic breeding sites such as pools, tires, tanks, and pots, among others; biological control through fish, and bacteria, such as the species *Bacillus thuringiensis* var *israelensis*; growth regulators and chemical control with the application of insecticides [14].

In Colombia, organophosphate, organochlorine, carbamate, and pyrethroid insecticides have been used to control *Ae. aegypti* for more than five decades as the main tool for the national and departmental programs for the prevention and control of vector-transmitted diseases (VTD) [15]. However, resistance to these insecticides has been increasingly recorded, which has made control actions difficult within VTD programs in different regions of the country [16]. In Colombia, pyrethroids have been one of the most widely used insecticides to control adult forms of *Ae. aegypti*, because of its low impact on the environment, low toxicity to mammals, and greater effectiveness. In La Guajira, through the Vector Control Program, malathion and temephos have been used to control dengue, chikungunya, and Zika outbreaks; however, as of 2018, alpha-cypermethrin, pirimiphos-methyl, and lambda-cyhalothrin have been used [17].

The target site of action of pyrethroids is the voltage-gated sodium channel (VGSC): a transmembrane protein made up of four homologous domains (I–IV), each with six hydrophobic segments (S1–S6) [18]. Conformational changes in the structure of this channel have been identified by mutations that confer resistance to *Ae. aegypti* to pyrethroids, and these are called kdr (knockdown resistance) mutations causing knockdown resistance [19]. In the Caribbean region of Colombia, resistance to the pyrethroids lambda-cyhalothrin, deltamethrin, cyfluthrin, and permethrin has been found in *Ae. aegypti* populations with the identification of the kdr mutations V1016I, F1534C, and V410L [20,21,22,23,24,25]. In La Guajira, few works have evaluated the susceptibility status of vector populations. However, resistance to permethrin, deltamethrin, cyfluthrin, and lambda-cyhalothrin has been identified in the municipality of San Juan del Cesar, whereas resistance to lambda-cyhalothrin has been identified in the district of Riohacha [21,23]. These findings have allowed the interdisciplinary team of the vector program to develop new strategies that consist of rotating insecticide molecules to control these arboviral diseases. Despite these results, the susceptibility of *Ae. aegypti* to pyrethroids and their underlying resistance mechanisms are unknown for most of the municipalities in this department. All the above information is essential, observing the endemic behavior of dengue and the introduction of chikungunya and Zika viruses in recent years in La Guajira. Likewise, it is important to consider that the susceptibility of insects to pyrethroids varies in space and time between populations according to the selection pressure exerted by these insecticides between the different municipalities.

The main objective of this study was to evaluate the resistance status of *Ae. aegypti* to the pyrethroids permethrin, deltamethrin, and lambda-cyhalothrin and to assess the presence and contribution of the kdr mutations V410L, V1016I, and F1534C to pyrethroid resistance in the 15 municipalities of the department of La Guajira, Colombia.

## 2. Materials and Methods

### 2.1. Study Area

**Figure 1:**
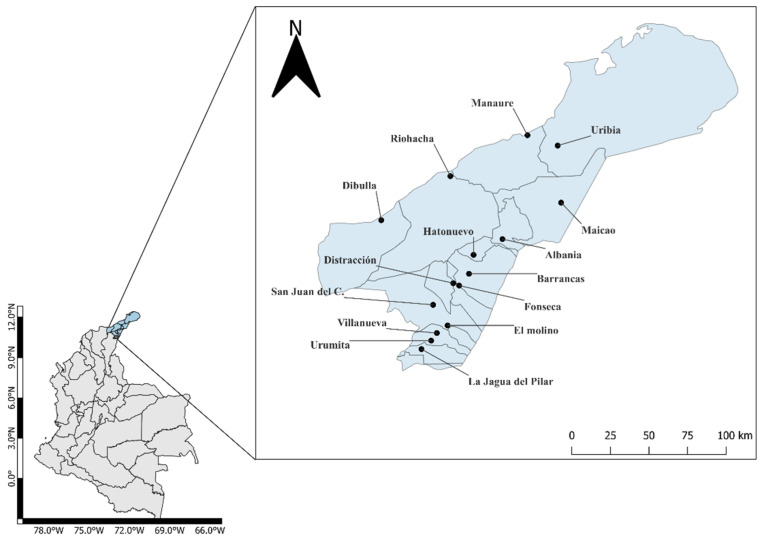
*Aedes aegypti* collection sites in the department of La Guajira, Colombia.

The study was carried out during 2020 and 2021 in the 15 municipalities of the department of La Guajira (Figure 1). La Guajira is bordered on the north by the Caribbean Sea, on the east by the Caribbean Sea and Venezuela, on the south by the department of Cesar, and on the west by the department of Magdalena and the Caribbean Sea. It has an area of 20,848 km^2^, representing 1.8% of the Colombian territory.

The inclusion criteria for the selection of the neighborhoods where the parental strains of *Ae. aegypti* were collected in the selected municipalities were high indices of *Ae. aegypti*, the frequent application of insecticides for the control of this mosquito, and a high incidence of dengue in the last ten years prior to the start of the project.

### 2.2. Collection of Mosquitoes and Obtaining F_1_/F_2_

Entomological inspections were carried out for the collection of immature forms of *Ae. aegypti* in potential breeding sites such as swimming pools, plastic/metallic bins, tires, and pots. The collected entomological material was transported in 500-mL plastic bottles to the insectarium of the Barranquilla Campus of Universidad Libre, where the F_1_ and F_2_ generations were obtained under controlled conditions of temperatures (28 ± 2 °C), relative humidity (60 ± 10%), and 12 h:12 h (light:dark) photoperiod.

### 2.3. Bioassays

The pyrethroids permethrin, deltamethrin, and lambda-cyhalothrin were evaluated using the stock solutions of the technical grade insecticides (ChemService, West Chester, PA, USA) using acetone as a diluent. The diagnostic doses (DD) of permethrin (15 µg/bottle), deltamethrin (10 µg/bottle), and lambda-cyhalothrin (10 µg/bottle) were used, considering the diagnostic time (DT) of 30 min for the three cases [26]. The bottle bioassay [27] used a 250-mL Schott bottle containing 1 mL of an acetone solution of technical-grade insecticide at the DD of each insecticide. The bottle was capped and shaken to ensure uniform coverage and then allowed to stand and dry for 24 h at room temperature with protection from light.

Between 15 and 25 unfed 3-day-old adult females were placed in the bottles until the diagnostic time was completed. The number of dead mosquitoes at the time of diagnosis was recorded to determine the level of susceptibility in the study populations. This procedure was repeated four times. A bottle with acetone, without insecticide, was used as a control bottle. In all cases, the correction was applied by Abbott’s formula when mortality in the control group was between 5 and 20%, and the bioassay was invalid when mortality exceeded 20% [28].

All the procedures described above were performed in all field populations of *Ae. aegypti* and the susceptible Rockefeller strain as a reference.

The frequency of resistance was calculated, and the populations were designated susceptible when mortality was ≥98%, while between 90 and 97% suggested the possibility of resistance that requires confirmation and mortality with <90% indicating resistance [29].

In the populations where resistance was determined at the diagnostic dose and time, the intensity of the resistance was analyzed through new bioassays that consisted of evaluating two (2×), five (5×), and ten (10×) times the DD [29]. The results were interpreted as follows, ≥98% mortality with 5× DD exposure was considered low-intensity resistance, and <98% mortality was considered moderate to high-intensity resistance. For the case of exposure to 10x the DD, a mortality ≥98% was considered moderate intensity resistance, while <98% mortality was considered high-intensity resistance.

A Kruskal–Wallis test followed by Dunn’s multiple comparisons test (α = 0.05) was used to compare the mortality rates produced by the DD of permethrin, deltamethrin, and lambda-cyhalothrin.

### 2.4. Identification of kdr Mutations

Forty mosquitoes of the F_0_ generation (field) were selected from each population and evaluated. DNA extraction was performed using the Extracta Kit (Quanta Biosciences 95091-250, Gaithersburg, MD, USA) and was later quantified using a UV-Vis NanoDrop One Microvolume spectrophotometer (Thermo Scientific; Waltham, MA, USA).

PCRs were performed in a CFX90 real-time thermal cycler (Bio-Rad; Hercules, CA, USA). The genotype for loci 1016, 1534, and 410 was determined by melting curve analysis. The amplification of the V1016I mutation in the VGSC gene was performed according to the method described by Saavedra et al. [30]; the final volume of each reaction was 20 µL, which included: 10 μL of Perfecta Sybr^®^ Green Supermix (Quanta 95054-500, Beverly, MA, USA), 1 μL of both primers V1016(r) (5′-CGGGCAGGGCGGCGGGGGCGGGGCCACAAATTGTTTCCCACCCGCACCGG-3′), I1016(f) (5′-GCGGGCACAATTGTTTCCCACCCGCACTGA-3′) and I1016(r) (5′-GGATGAACCGAAATTGGACAAAAGC-3′), 6 µL of water, and 1 µL of the DNA template. Amplification reactions were performed according to the following thermal profile: an initial denaturation cycle at 95 °C for 3 min, 40 cycles at 95 °C for 10 s, 60 °C for 10 s, and 72 °C for 30 s. A final extension was performed at 95 °C for 10 s. The melting curves were determined by a denaturation gradient from 65 °C to 95 °C with an increase of 0.2 °C every 10 s.

The F1534C mutation was identified according to the method described by Yanola et al. [31]; the final volume of each reaction was 20 μL, which included 9 μL of Perfecta Sybr^®^ Green Supermix (Quanta 95054-500, Beverly, MA, USA), 0.66 μL of the primers F1534(f) (5′-GCGGGCTCTACTTTGTGTTCTTCATCATATT-3′) and F1534(r) (5′-TCTGCTCGTTGAAGTTGTCGAT-3′), 0.65 μL of primer C1534(f) (5′-GCGGGCAGGGCGGCG GGGGCGGGGCCTCTACTTTGTGTTCTTCATCATGTG-3′), 7.5 µL of water, and 2 µL of the DNA template. Amplification reactions were performed with the following thermal profile: an initial denaturation cycle at 95 °C for 3 min, 37 cycles at 95 °C for 10 s, 57 °C for 30 s and 72 °C for 30 s, and one final extension at 95 °C for 10 s. The melting curves were determined using a denaturation gradient from 65 to 95 °C with an increase of 0.5 °C every 5 s.

The V410L mutation was identified according to Hadii et al. [32] with a final volume of 21 μL containing 10 μL of Perfecta Sybr^®^ Green Supermix (Quanta 95054-500, Beverly, MA, USA), 0.1 μL of each of the primers L410(f) (5′-GCGGGCATCTTCTTGGGTTCGTTCTACCATT-3′) and V410(f) (5′-GCGGGCAGGGCGGCGGGGGCGGGGCCATCTTCTTGGGTTCGTTCTACCGTG-3′), 0.2 μL of primer L410(r) (5′-TTCTTCCTCGGCGGCCTCTT-3′), 9.6 μL of water, and 1.0 μL of the DNA template. Amplification reactions were performed with the following thermal profile: an initial denaturation cycle at 95 °C for 3 min, followed by 39 cycles at 95 °C for 10 s, 95 °C for 10 s, and 60 °C for 10 s. The melting curves were determined by a denaturation gradient from 65 to 95 °C with an increase of 0.2 °C every 10 s.

Homozygous-recessive (mutant) mosquitoes for the V1016I, F1534C, and V410L mutations were used as positive controls.

The melting curve results for the V1016I mutation were interpreted as follows. A single peak at 83 °C meant a dominant homozygous mosquito (V/V), that is, a susceptible genotype. A single peak at 76 °C corresponded to a homozygous recessive (I/I) mosquito, that is, a resistant genotype. Finally, the presence of the two peaks at 76 and 83 °C corresponded to a heterozygous mosquito (V/I). The melting curve results for the F1534C mutation were interpreted as follows. A single peak at 80 °C corresponded to a homozygous dominant mosquito (F/F), that is, a susceptible genotype. A single peak seen at 85 °C corresponded to a homozygous recessive (C/C) mosquito, that is, a resistant genotype. Finally, the presence of the two peaks at 80 and 85 °C meant a heterozygous mosquito (F/C). The melting curve results for the V410L mutation were interpreted as follows. A peak at 86.5 °C corresponded to a dominant homozygote (V/V); a peak at 83 °C corresponded to a recessive homozygote (L/L), susceptible and resistant genotypes, respectively. A peak at 83 °C and another at 86.5 °C corresponded to a heterozygote (V/L).

### 2.5. Spatial Distribution of Resistance to Pyrethroids

The insecticide resistance distribution was analyzed using the inverse distance weighting (IDW) interpolation procedure. The distance between the sampled sites and the mortality for each pyrethroid was used to estimate the mortality in the unsampled regions. IDW interpolation relies on the assumption that closer points have more similar values than locations separated by greater distances [33]. This association between distance and feature value is regulated by the power parameter in the IDW formula. To select the power value that gives the best estimates of predicted mortalities, the root squared mean error (RSME) was calculated by comparing true data values to interpolated values. Four different values (2, 2.5, 3, 3.5, and 4) were tested, and the power value with the lower RMSE was selected as it showed the lowest difference between the interpolated and true values. All analyses were conducted in R version 4.1.0 and QGIS version 3.24.

Allele and genotype frequencies were calculated; we verified that the populations were in Hardy–Weinberg equilibrium using a χ^2^ test. Wright’s F_IS_ inbreeding coefficient was estimated, along with Wald’s correction [34,35]. In addition, the frequencies of the tri-locus haplotypes in the study populations were determined.

To assess whether the resistant haplotypes in *Ae. aegypti* were related to the frequency and intensity of resistance, a Spearman’s rank correlation analysis (α = 0.05) was performed between its frequency and the mortality rate caused by pyrethroid insecticides.

## 3. Results

### 3.1. Frequency and Intensity of Pyrethroid Resistance

**Figure 2:**
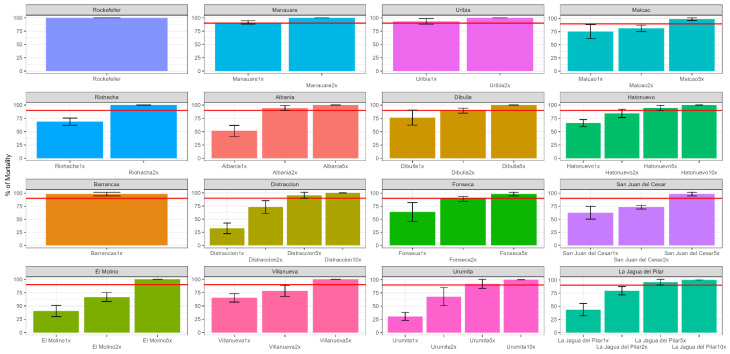
Mortality (with standard deviations) of *Ae. aegypti* following exposure to 1× DD, 2× DD, 5× DD, and 10× DD of permethrin in bottle bioassays. The red lines indicate the resistance threshold (90% mortality).

The frequency of mortality obtained after exposure for 30 min to permethrin DD was less than 90% in 12 of the 15 populations analyzed, with mortality ranging from 30 to 76% (Figure 2). Only the populations from Manaure and Uribia showed values of 92 and 97% mortality, respectively, and the population of Barrancas exhibited 98% mortality showing susceptibility for the three pyrethroids.

**Figure 3:**
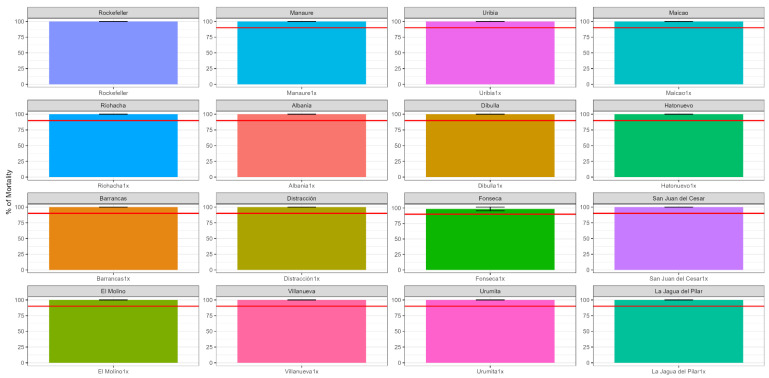
Mortality (with standard deviation) of *Ae. aegypti* following exposure to 1× DD of deltamethrin in bottle bioassays. The red lines indicate the resistance threshold (90% mortality).

All populations showed susceptibility to deltamethrin with 99–100% mortality (Figure 3).

**Figure 4:**
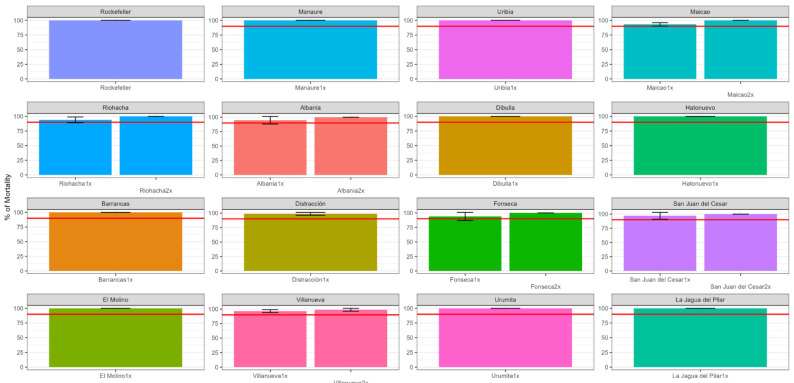
Mortality (with standard deviation) of *Ae. aegypti* following exposure to 1× DD and 2× DD of lambda-cyhalothrin in bottle bioassays. The red lines indicate the resistance threshold (90% mortality).

In the case of lambda-cyhalothrin, the mortality frequency for all the populations was greater than the 90% threshold, suggesting the beginning of the emergence of resistance for the populations of Albania, Fonseca, Maicao, Riohacha, San Juan del Cesar, and Villanueva since they showed mortalities between 93 and 97%. The rest of the populations showed susceptibility to this insecticide (Figure 4).

When analyzing the intensity of resistance to permethrin, 100% mortality was evidenced at 2× DD in the populations from Manaure, Riohacha, and Uribia; only the Albania population showed 94% mortality, and the rest of the populations displayed mortality fluctuating between 68 and 90%. The populations from Distracción, Hatonuevo, La Jagua del Pilar, and Urumita showed a moderate intensity of resistance with mortalities of 92–96% when exposed to 5× DD. In contrast, for this same insecticide, 100% mortality was observed at the concentration of 10× in these same populations (Figure 2).

Regarding the intensity of resistance to lambda-cyhalothrin, mortalities between 99 and 100% were observed at the 2× dose in the populations from Albania, Fonseca, Maicao, Riohacha, San Juan del Cesar, and Villanueva (Figure 4).

**Figure 5:**
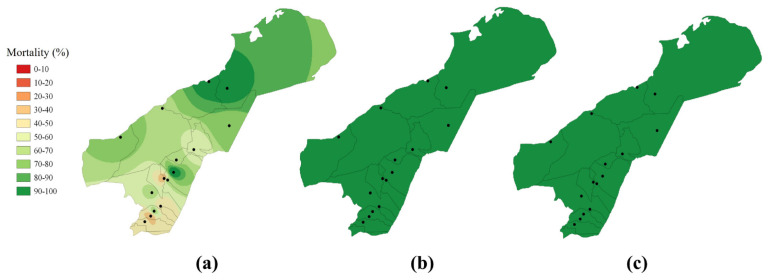
Distribution of pyrethroid resistance (% of mortality to the DD) to (**a**) permethrin, (**b**) deltamethrin, and (**c**) lambda–cyhalothrin in *Ae. aegypti* populations from La Guajira, Colombia using IDW interpolation.

The populations showed a higher frequency of resistance to permethrin than deltamethrin and lambda-cyhalothrin (Figure 5). The trends in mortality were significantly different between permethrin and deltamethrin and between permethrin and lambda-cyhalothrin (*p* < 0.01). Power values of 2, 3.5, and 4 resulted in lower RSME values for lambda-cyhalothrin, permethrin, and deltamethrin, respectively. IDW interpolation (Figure 5) showed that deltamethrin and lambda-cyhalothrin shared the same pattern where predicted mortalities were located between 90 and 100%. However, for permethrin, northern locations displayed higher predicted mortalities than southern populations, where the predicted values could be below 50%.

### 3.2. Allele and Genotype Frequencies of the kdr Mutations V410L, V1016I, and F1534C

Six hundred mosquitoes from all populations (40/population) were genotyped for V410L, V1016I, and F1534C mutations. For the V410L mutations, three genotypes, VV 410, VL 410, and LL 410, were detected in each field population, except for the populations from El Molino and Villanueva, where the LL 410 genotype was not found. The L 410 mutant allele was more frequent in the population from Uribia, with 0.61, and the least frequent in the population from El Molino 0.10. All populations were found to be in a Hardy–Weinberg equilibrium except for the Manaure population. An excess of heterozygotes was evidenced in the populations from Barrancas, Dibulla, El Molino, La Jagua del Pilar, Manaure, and San Juan del Cesar (Table 1).

For the V1016I mutation, the three genotypes VV 1016, VI 1016, and II 1016 were detected in each population, except for the populations from El Molino and Urumita, where genotype II 1016 was not observed. The I1016 mutant allele was the most frequent in the population from Uribia, with 0.61, and the least frequent in the population from El Molino 0.09. All populations were found to be in the Hardy–Weinberg equilibrium, except for the Hatonuevo and Urumita populations, and an excess of heterozygotes was evidenced in the populations from Barrancas, El Molino, La Jagua del Pilar, Maicao, Manaure, San Juan del Cesar, Urumita, and Villanueva (Table 2).

Regarding the F1534C mutation, the CC 1534 genotype was found in all the populations examined. The allelic frequency for the populations from Manaure, Distracción, Riohacha, and Albania ranged between 0.91 and 0.95, and for the population from Villanueva, an allele frequency of 0.69 was observed. The mutation was found to be fixed in the population from Uribia. Most of the populations were in the Hardy–Weinberg equilibrium, except those from Dibulla, Distraction, and Uribia. On the other hand, the populations from Albania, Dibulla, Fonseca, Hatonuevo, Maicao, Manaure, Urumita, and Villanueva showed an excess of heterozygotes (Table 3).

**Figure 6:**
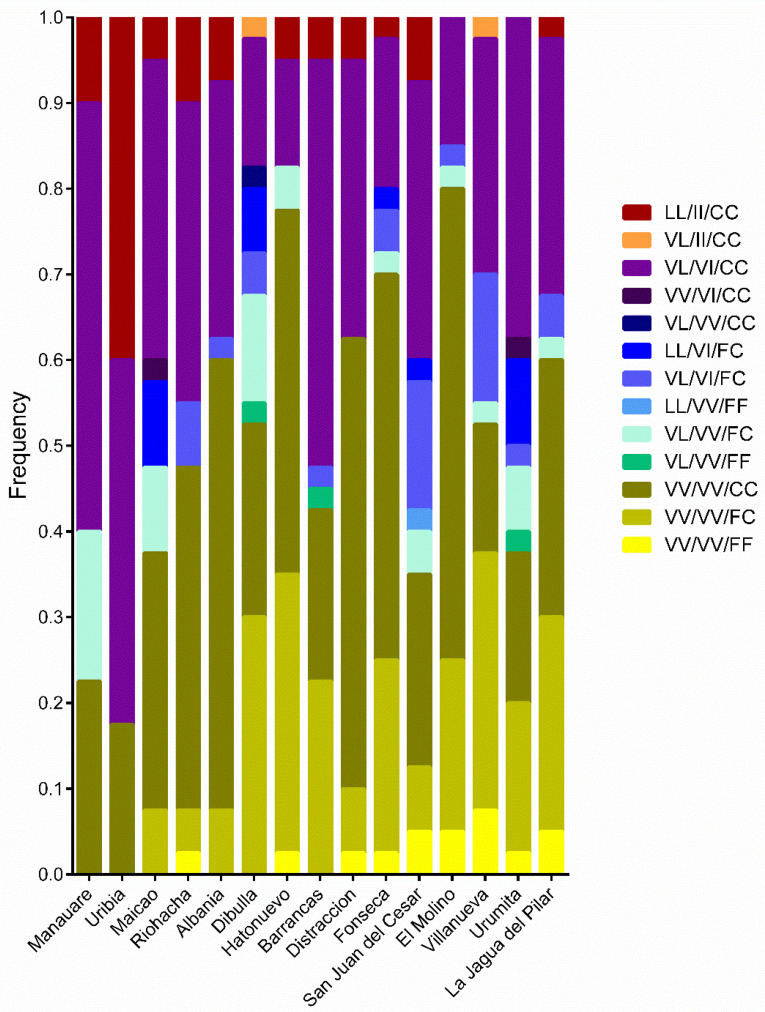
Frequencies of tri-locus haplotypes in each population of *Ae. aegypti* from La Guajira, Colombia. Haplotype order: 410/1016/1534.

A total of 13 tri-locus combinations were detected among the populations (Figure 6). The homozygous triple-resistant haplotype (II1016/CC1534/LL410) occurred in 11 populations with very low frequencies. The total frequency was 6.6% of the total number of mosquitoes analyzed. Regarding the co-occurrence of kdr mutations, a total of triple-homozygous mutants was found in 6.6% of the 600 mosquitoes analyzed, where this haplotype was found in the populations from Albania (7.5%), Barrancas (5%), Distracción (5%), Fonseca (2.5%), Hatonuevo (5%), La Jagua del Pilar (2.5%), Maicao (5%), Manaure (10%), Riohacha (10%), San Juan del Cesar (7.5%), and Uribia (40%).

The most frequent haplotypes were VV410/VV1016/CC1534, VL410/VI1016/CC1534, and VV410/VV1016/FC1534, with 32, 31, and 16% of the total mosquitoes, respectively (Figure 6).

When analyzing the association of the mortality rate when exposing the mosquitoes to 1× DD with the frequencies of the tri-locus haplotypes for the three insecticides, a significant positive association was only found for permethrin with the VL410/VV1016/CC1534 haplotype (r = 0.217, *p* < 0.01). The same was seen in the case of deltamethrin, with the maximum association value for the VL410/VI1016/CC1534 haplotype (r = 0.25, *p* < 0.01), and in the case of lambda-cyhalothrin, with the maximum association value for the VL/VV/CC haplotype (r = 0.24, *p* < 0.01). Frequencies of the triple-resistant haplotype (LL410/II1016/CC1534) were significantly associated with mortality rates when the mosquitoes were exposed to 2X DD permethrin (r = 0.66, *p* < 0.05).

## 4. Discussion

Due to the selection pressure exerted by lambda-cyhalothrin and deltamethrin in Colombia, resistance to these and other pyrethroid-type molecules has been reported in different populations of *Ae. aegypti* [15]. Specifically, in the department of La Guajira, there has been a history of resistance to lambda-cyhalothrin, deltamethrin, and permethrin in the populations from San Juan del Cesar and for lambda-cyhalothrin in Riohacha [21,23]. The results found in this work are consistent with these previous studies, except for the susceptibility result found for deltamethrin in the population from San Juan del Cesar. This may be due to the fact that low resistance to deltamethrin had previously been reported in this population through the determination of the resistance factor by a lethal concentration 50 (LC_50_). By contrast, in this work, susceptibility was determined through DD [21].

On the other hand, the results of the present work constitute the first record that expands the knowledge of the susceptibility status of the thirteen remaining populations in the department of La Guajira, generally finding resistance to permethrin, the populations in danger of developing resistance to lambda-cyhalothrin, and susceptibility to deltamethrin, except in the population of Barrancas, which exhibited susceptibility to the three insecticides. Resistance to lambda-cyhalothrin has also been widely reported in Colombia in the departments of Chocó, Antioquia, Putumayo, Atlántico, Cesar, La Guajira, Sucre, Córdoba, Bolívar, Cundinamarca, Santander, Caquetá, Guaviare, Meta, and Casanare [21,23,36,37,38,39]. For deltamethrin in Colombia, most *Ae. aegypti* populations have been reported as susceptible; there is also a record of resistance in populations from the departments of Bolívar, Cesar, Córdoba, Atlántico, Cundinamarca, Caquetá, and Casanare [21,36,39]. This variability in the status of susceptibility to lambda-cyhalothrin and deltamethrin may be due to the difference in the selection pressure exerted with these insecticides, considering the differences in the incidence of dengue in Colombia, if deemed that there is a heterogeneous pattern in the transmission of this disease in the country when finding hyperendemic, endemic, and hypoendemic areas [40]. In addition, it is important to note that the use of insecticides in agriculture is low for the department of La Guajira, considering that most of the rural land use is for livestock production (82%) and only approximately 9% is for agricultural production [41]. However, from 1960 to the late 1980s, the Cesar River Valley, south of the department of La Guajira, was the main cotton district in Colombia; DDT, chlorpyrifos, and methomyl were the main insecticides used for pest control [42].

The resistance to permethrin found in the populations evaluated has also been recorded in other populations of the vector in Colombia [25,37,39]. It may be due to cross-resistance with other pyrethroid-type molecules such as lambda-cyhalothrin and/or organochlorine DDT, considering that in Colombia, this insecticide was suspended at the beginning of the 1990s by Resolution 10255 of the Health Ministry [21]. Cross-resistance has also been reported with other insecticides, such as alpha-cypermethrin, in populations resistant to pyrethroids and DDT in Colombia [24]. All this evidence demonstrates the importance of evaluating over time both the insecticides that have been applied to control this species, as well as other insecticides not used by government agencies, to contribute to the prevention of resistance and provide other alternatives for vector control.

Regarding the spatial distribution of insecticide resistance, we showed the resistance profile for three pyrethroids in unsampled regions within La Guajira using IDW interpolation. It is noteworthy that permethrin was the only insecticide with mortalities below 90% for southern areas. However, all results must be interpreted with caution, considering the limitations of the interpolation technique. Although highly implemented, the IDW method is a deterministic procedure that does not allow the calculation of variances of predicted, unmeasured sites. Additionally, this procedure relies on the distance between the sampled locations and the non-variable power value to predict the values of unmeasured sites [43]. It is also important to mention that other factors besides distance (e.g., insecticide application and mosquito population dynamics) influence the distribution of insecticide resistance.

Pyrethroid resistance is mainly associated with the presence of kdr mutations in the para gene of VGSC in insects such as *Ae. aegypti*. In this species, the kdr mutations G923V, L982W, I1011M, V1016G, I1011V, V1016I, F1534C, S989P, D1763Y, T1520I, and V410L have been identified worldwide with differences in allele and genotype frequencies between *Ae. aegypti* populations reported [30,31,44,45,46,47,48].

In the Americas, the V1016I, F1534C, and V410L mutations have been mainly associated with resistance to pyrethroids in populations in Mexico [30,49,50,51], Brazil [32,52,53], Colombia [21,23,24,25], Perú [54], Costa Rica [55], Puerto Rico [56], and Venezuela [57].

These mutations have also been reported and associated with resistance in populations of this vector in Colombia and in the departments of Antioquia, Valle del Cauca, Atlántico, Cesar, Bolívar, Magdalena, Sucre, Córdoba, Meta, Santander, and Quindío, with allele frequencies for V1016I between 0.02 and 0.72, for F1534C between 0.44 and 1.0, and for V410L between 0.05 and 0.72 [21,22,23,24,25,58,59]. Specifically, in the department of La Guajira, the kdr mutations identified were V1016I and F1534C in the San Juan del Cesar populations and V1016I, F1534C, and V410L in Riohacha [21,23,24]. In the case of the San Juan del Cesar population, allele frequencies of 0.28 and 0.77 have been reported for I1016 and C1534, respectively. By contrast, in the present study, these frequencies increased to 0.33 and 0.78, respectively. In the Riohacha population, allele frequencies were 0.25 for I016 and 0.71 for C1534, while in the present study, there was an increase between 0.31 and 0.91, respectively.

Regarding the allele frequency for L410 in a population of this same municipality, a frequency of 0.36 was determined, and we found in the present study a decrease to 0.31 [21,23,24]. The other populations had no history of identifying kdr mutations, so the results of this work constitute the first record for these populations, in which allele frequencies for L410 were between 0.10 and 0.61, for I1016 between 0.15 and 0.60, and for C1534 between 0.68 and 1.0. It is important to highlight that the highest frequency for the mutations was identified in the Uribia population, which is an isolated population from a geographical point of view in the upper Guajira that corresponds to a desert area and has been subjected in recent years to high selection pressure due to the increase in dengue incidence and the introduction of chikungunya and Zika viruses.

For the department of La Guajira, there are only two previous studies on the co-occurrence of kdr mutations in populations of *Ae. aegypti*; when comparing the results of these works with those obtained in the present study, an increase in the frequency of the double-homozygous mutant (II/CC) haplotype was observed in the municipality of San Juan del Cesar [24] and triple-homozygous mutant (II/CC/LL) in the Riohacha district [23]. Considering the results obtained, the evident resistance to permethrin, and the risk of developing resistance to lambda-cyhalothrin, the vector control program in La Guajira recommends malathion, pirimiphos-methyl, and deltamethrin for adult control [17].

## 5. Conclusions

The populations from La Guajira, Colombia, were resistant to permethrin with moderate intensity, associated with the frequency of the triple-mutated haplotype (LL410/II1016/CC1534), and were susceptible to deltamethrin and most to lambda-cyhalothrin, with six populations at risk of developing resistance.

## Figures and Tables

**Table 1:** Allele and genotype frequencies of V410L in populations of *Ae. aegypti* from the department of La Guajira, Colombia.

Population	n	VV	VL	LL	Frequency (IC 95%)	F_IS_	x^2^ Hardy-Weinberg	*p* Value
Manaure	40	9	27	4	0.44 (0.30–0.59)	−0.37	5.51	0.02
Uribia	40	7	17	16	0.61 (0.46–0.75)	0.10	0.43	0.50
Maicao	40	16	18	6	0.38 (0.24–0.53)	0.04	0.06	0.80
Riohacha	40	19	17	4	0.31 (0.20–0.47)	0.01	0	0.94
Albania	40	24	13	3	0.24 (0.13–0.39)	0.10	0.42	0.51
Dibulla	40	20	17	3	0.29 (0.17–0.44)	−0.03	0.05	0.81
Hatonuevo	40	31	7	2	0.14 (0.06–0.28)	0.26	2.74	0.10
Barrancas	40	17	21	2	0.31 (0.20–0.47)	−0.22	1.96	0.16
Distracción	40	24	13	3	0.24 (0.13–0.39)	0.10	0.42	0.51
Fonseca	40	28	10	2	0.18 (0.09–0.32)	0.13	0.72	0.40
San Juan del Cesar	40	14	21	5	0.39 (0.25–0.54)	−0.10	0.44	0.50
El Molino	40	32	8	0	0.10 (0.04–0.24)	−0.11	0.49	0.50
Villanueva	40	21	19	0	0.24 (0.13–0.39)	−0.31	3.88	0.05
Urumita	40	16	20	4	0.35 (0.22–0.50)	−0.09	0.39	0.53
La Jagua del Pilar	40	24	15	1	0.21 (0.11–0.36)	−0.12	0.58	0.44

Abbreviations: n, sample size; VV, wild type; VL, heterozygotes; LL, homozygotes resistant.

**Table 2:** Allele and genotype frequencies of V1016I in populations of *Ae. aegypti* from La Guajira, Colombia.

Population	n	VV	VI	II	Frequency (IC 95%)	F_IS_	x^2^ Hardy-Weinberg	*p* Value
Manaure	40	16	20	4	0.35 (0.22–0.50)	−0.1	0.39	0.53
Uribia	40	7	17	16	0.61 (0.46–0.75)	0.1	0.44	0.51
Maicao	40	19	19	2	0.29 (0.17–0.44)	−0.16	1.02	0.31
Riohacha	40	19	17	4	0.31 (0.19–0.47)	0.01	0	0.94
Albania	40	24	13	3	0.24 (0.13–0.39)	0.1	0.42	0.52
Dibulla	40	28	11	1	0.16 (0.08–0.31)	0	0	1
Hatonuevo	40	33	5	2	0.11 (0.05–0.25)	0.37	5.59	0.01
Barrancas	40	18	20	2	0.30 (0.18–0.45)	−0.19	1.45	0.23
Distracción	40	25	13	2	0.21 (0.11–0.36)	0.02	0.03	0.86
Fonseca	40	29	10	1	0.15 (0.07–0.29)	0.02	0.02	0.9
San Juan del Cesar	40	17	20	3	0.33 (0.20–0.48)	−0.14	0.78	0.38
El Molino	40	33	7	0	0.09 (0.03–0.21)	−0.096	0.36	0.55
Villanueva	40	22	17	1	0.24 (0.13–0.39)	−0.17	1.2	0.27
Urumita	40	19	21	0	0.26 (0.15–0.42)	−0.35	5.06	0.02
La Jagua del Pilar	40	25	14	1	0.20 (0.10–0.35)	−0.09	0.35	0.55

Abbreviations: n, sample size; VV, wild type; VI, heterozygotes; II, homozygotes resistant.

**Table 3:** Allele and genotype frequencies of F1534C in populations of *Ae. aegypti* from La Guajira, Colombia.

Population	n	FF	FC	CC	Frequency (IC95%)	F_IS_	x^2^ Hardy-Weinberg	*p* Value
Manaure	40	0	7	33	0.91 (0.79–0.97)	−0.1	0.37	0.54
Uribia	40	0	0	40	1.00 (0.91–1.00)	-	-	-
Maicao	40	0	11	29	0.86 (0.72–0.94)	−0.16	1.02	0.31
Riohacha	40	1	5	34	0.91 (0.79–0.97)	0.22	2.05	0.15
Albania	40	0	4	36	0.95 (0.84–0.99)	−0.05	0.11	0.74
Dibulla	40	1	22	17	0.70 (0.55–0.82)	−0.37	5.4	0.02
Hatonuevo	40	1	15	24	0.79 (0.64–0.89)	−0.12	0.58	0.44
Barrancas	40	1	10	29	0.85 (0.71–0.93)	0.02	0.02	0.9
Distracción	40	1	3	36	0.94 (0.82–0.98)	0.36	5.18	0.02
Fonseca	40	1	13	26	0.81 (0.67–0.90)	−0.07	0.18	0.67
San Juan del Cesar	40	3	12	25	0.78 (0.63–0.88)	0.14	0.78	0.38
El Molino	40	2	10	28	0.83 (0.69–0.91)	0.13	0.72	0.40
Villanueva	40	3	19	18	0.69 (0.53–0.81)	−0.11	0.44	0.5
Urumita	40	2	15	23	0.76 (0.61–0.87)	−0.03	0.05	0.82
La Jagua del Pilar	40	2	13	25	0.79 (0.64–0.89)	0.02	0.03	0.85

Abbreviations: n, sample size; FF, wild type; FC, heterozygotes; CC, homozygotes resistant.

## Data Availability

All the required data relevant to the presented study are included in the manuscript.
